# Analysis of density based and fuzzy c-means clustering methods on lesion border extraction in dermoscopy images

**DOI:** 10.1186/1471-2105-11-S6-S26

**Published:** 2010-10-07

**Authors:** Sinan Kockara, Mutlu Mete, Bernard Chen, Kemal Aydin

**Affiliations:** 1 Computer Science Department, University of Central Arkansas, Conway, AR, USA; 2 Department of Computer Science, Texas A&M University-Commerce, Commerce, TX, USA; 3 Computer Science Department, University of Arkansas at Pine Bluff, Pine Bluff, AR, USA

## Abstract

**Background:**

Computer-aided segmentation and border detection in dermoscopic images is one of the core components of diagnostic procedures and therapeutic interventions for skin cancer. Automated assessment tools for dermoscopy images have become an important research field mainly because of inter- and intra-observer variations in human interpretation. In this study, we compare two approaches for automatic border detection in dermoscopy images: density based clustering (DBSCAN) and Fuzzy C-Means (FCM) clustering algorithms. In the first approach, if there exists enough density –greater than certain number of points- around a point, then either a new cluster is formed around the point or an existing cluster grows by including the point and its neighbors. In the second approach FCM clustering is used. This approach has the ability to assign one data point into more than one cluster.

**Results:**

Each approach is examined on a set of 100 dermoscopy images whose manually drawn borders by a dermatologist are used as the ground truth. Error rates; false positives and false negatives along with true positives and true negatives are quantified by comparing results with manually determined borders from a dermatologist. The assessments obtained from both methods are quantitatively analyzed over three accuracy measures: border error, precision, and recall.

**Conclusion:**

As well as low border error, high precision and recall, visual outcome showed that the DBSCAN effectively delineated targeted lesion, and has bright future; however, the FCM had poor performance especially in border error metric.

## Introduction

Melanoma is the fifth most common malignancy in the United States and has rapidly become one of the leading cancers in the world. Malignant melanoma is the most deadly form of skin cancer and the fastest growing skin cancer type in the human body. 8,441 deaths out of 68,720 incidences are estimated numbers in the United States in 2009 [1]. If it is detected early, melanoma can often be cured with a simple excision operation. Dermoscopy is the major non-invasive skin imaging technique that is extensively used in the diagnosis of melanoma and other skin lesions. Dermoscopy improves upon simple photography by revealing more of the subsurface structures underneath the skin, and is now widely used by dermatologists. The contact dermoscopy technique consists of placing fluid such as mineral oil, water, or alcohol on the skin lesion that is subsequently inspected using a digital camera and a hand-held dermoscopy attachment such as Dermlite. The fluid placed on the lesion eliminates surface reflection and renders the cornified layer translucent; thus, allowing a better visualization of pigmented structures within the epidermis, the dermoepidermal junction and the superficial dermis. 

For early detection of melanoma, finding lesion borders is the first and key step of the diagnosis since the border structure provides life saving information for accurate diagnosis. Currently, dermatologists draw borders manually which is described as *tedious* and time consuming. That is where *computer-aided border detection* of dermoscopy images comes in to the picture. Detecting melanoma early is critical because the melanoma not detected early can be fatal. Also, speed is critical because of a lack of dermatologists to screen all the images. Thus, physician error increases with rapid evaluation of cases [2]. For these very reasons, automated systems would be a significant help for dermotologists. The proposed method in this study is a fully automated system. By fully automated authors mean that prior to and during the processing there is no human intervention in the system. The purpose with this system is to increase dermotologist’s comfort level with his/her decision; however, a dermatologist always constitutes the final decision on the subject.

## Background

Differentiating or partitioning objects from background or other objects on an image is called image segmentation. Solutions to the image segmentation have widespread applications in various fields; including medical diagnosis and treatment. Plenty of methods have been generated for grayscale and color image segmentations [3-6]. Four popular approaches [7] for image segmentation are: edge-based methods, threshold techniques [8], neighborhood-based techniques, graph-based methods [9,10], and cluster-based methods. Edge based techniques investigate discontinuities in image whereas neighborhood-based methods examine the similarity (neighborhoods) among different regions. Threshold methods identify different parts of an image by combining peaks and valleys of 1D or 3D histograms (RGB). Also, there exists numerous innovative graph-based image segmentation approaches in the literature. Shi et al. 1997-1998 [9,10] treated segmentation as a graph partitioning problem, and proposed a novel unbiased measure for segregating subgroups of a graph, known as the Normalized Cut criterion. More recently, Felzenszwalb et al. [11] developed another segmentation technique by defining a predicate for the existence of boundaries between regions, utilizing graph-based representations of images. In this study; however, we focus on cluster-based segmentation methods. In cluster-based methods, individual image pixels are considered as general data samples and assumed correspondence between homogeneous image regions and clusters in the spectral domain. 

Dermoscopy involves optical magnification of the region-of-interest, which makes subsurface structures more easily visible when compared to conventional macroscopic images [12]. This in turn improves screening characteristics and provides greater differentiation between difficult lesions such as pigmented Spitz nevi and small, clinically equivocal lesions [13]. However, it has also been demonstrated that dermoscopy may actually lower the diagnostic accuracy in the hands of an inexperienced dermatologists [14]. Therefore, novel computerized image understanding frameworks are needed to minimize the diagnostic errors that result from the difficulty and subjectivity of visual interpretations [15,16].

For melanoma investigation, delineation of region-of-interest is the key step in the computerized analysis of skin lesion images for many reasons. First of all, the border structure provides important information for accurate diagnosis. Asymmetry, border irregularity, and abrupt border cutoff are a few of many clinical features calculated based on the lesion border. Furthermore, the extraction of other important clinical indicators such as atypical pigment networks, globules, and blue-white vein  areas critically depends on the border detection [17].  The blue-white veil is described as an irregular area with blended blue pigment with a ground glass haze (white), as if the image were out of focus.

At the first stage for analysis of dermoscopy images, automated border detection is usually being applied [16]. There are many factors that make automated border detection challenging e.g. low contrast between the surrounding skin and the lesion, fuzzy and irregular lesion border, intrinsic artifacts such as cutaneous features (air bubbles, blood vessels, hairs, and black frames) to name a few [17]. According to Celebi et al. 2009 [17], automated border detection can be divided into four sections: pre-processing, segmentation, post-processing, and evaluation. Pre-processing step involves color space transformations [18], [19], contrast enhancement [20][21], and artifacts removal [22], [23], [24-28]. Segmentation step involves partitioning of an image into disjoint regions [29], [28],[23]. Post-processing step is used to obtain the lesion border [16], [30]. Evaluation step involves dermatologists’ evaluations on the border detection results. 

Regarding boundary of clusters, Lee and Castro [31] introduced a new algorithm of polygonization based on boundary of resulting point clusters. Recently Nosovskiy et al. [32] used another theoretical approach to find boundary of clusters in order to infer accurate boundary between close neighboring clusters. These two works principally study boundaries of finalized data groups (clusters). Schmid et al. [23] proposed an algorithm based on color clustering. First, a two-dimensional histogram is calculated from the first two principal components of the CIE L*u*v* color space. The histogram is then smoothed and initial cluster centers are obtained from the peaks using a perceptron classifier. At the final step, the lesion image is segmented. 

In this study for computer-aided border detection we use two clustering algorithms density based clustering (DBSCAN) [33] and multi level fuzzy C means clustering (FCM) and compare their performances over dermoscopy images for border detection. In the context of dermoscopic images, clustering corresponds to finding whether each pixel in an image belongs to skin lesion border or not. Automatic border detection makes dermatologist’s tedious manual border drawing procedure faster and easier.

## DBSCAN

With the aim of separating background from skin lesion to target possible melanoma, we cluster pixels of thresholded images by using DBSCAN. It takes a binary (segmented) image, and delineates only significantly important regions by clustering. The expected outcome of this framework is desired boundary of the lesion in a dermoscopy image.

**Figure 1:**
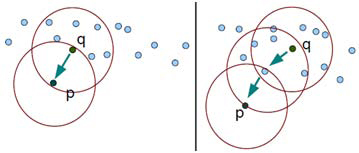
Direct density reachable (left) and density reachable property  of DBSCAN (right)

Technically, it is appropriate to tailor density based algorithm in which cluster definition guarantees that the number of positive pixels is equal to or greater than minimum number of pixels (MinPxl) in certain neighborhood of core points. The core point is that the neighborhood of a given radius (Eps) has to contain at least a minimum number of positive pixels (MinPxl), i.e., the density in the neighborhood should exceed pre-defined threshold (MinPxl). The definition of a neighborhood is determined by the choice of a distance function for two pixels p and q, denoted by dist(p,q). For instance, when the Manhattan distance is used in 2D space, the shape of the neighborhood would be rectangular. Note that DBSCAN works with any distance function so that an appropriate function can be designed for some other specific applications. DBSCAN is significantly more effective in discovering clusters of arbitrary shapes. It was successfully used for synthetic dataset as well as earth science, and protein dataset. Theoretical details of DBSCAN are given in [33]. Once the two parameters Eps and MinPxl are defined, DBSCAN starts to cluster data points (pixels) from an arbitrary point q as illustrated in Figure 1. 

Let I be a subimage that is of dimension N × N. For a pixel p, let p_x_ and p_y_ denote its position where top-left corner is (0, 0) of I. Let c_xy_ represent the color at (p_x_, p_y_).  The Eps-neighborhood of a pixel p, denoted by *NEps*(*p*), is defined by *NEps*(*p*) = {*q* ∈ *I* | *dist*(*p*, *q*) ≤ *Eps*} where dist is Euclidean distance. There can be found two kinds of pixels in a cluster: 1) pixels inside of the cluster (core pixels) and 2) pixels on the border of the cluster (border pixels). As expected, a neighborhood query for a border pixel returns notably less points than a neighborhood query of a core pixel. Thus, in order to include all points belonging to the same segment, we should set the minimum number of pixels (MinPxl) to a comparatively low value. This value, however, would not be characteristic for the respective cluster - particularly in the presence of negative pixels (non-cluster). Therefore, we require that for every pixel p in a cluster C there is a pixel q in C so that p is inside of the Eps-neighborhood of q and NEps(q) contains at least MinPxl pixels: | *NEps*(*p*) | ≥ *MinPxl* and *dist*(*p*, *q*) ≤ *Eps*. A pixel p is called density-reachable from a pixel q when there is a chain of pixels p_1_, p_2_, .., p_n_, where p_1_ = q, p_n_ = p. This is illustrated in Figure 1. A cluster C (segment) in image is a non-empty subset of pixels and given as:

*C* = {*p* ∩ *q* | | *NEps*(*p*) | ≥ *MinPxl* ,

where q is density reachable from p. DBSCAN centers around the key idea: to form a new cluster or grow an existing cluster the Eps-neighborhood of a point should contain at least a minimum number of points (MinPxl). 

**Algorithm 1** DBSCAN

DBSCAN (SubImage, Eps, Minpxl)

ClusterId:=nextId(NOISE);

FOR I FROM 1 To SubImage.height DO

FOR I FROM 1 To SubImage.width DO

Point := SubImage.get(i,j);

IF point.Cid = UNCLASSIFIED AND Point.positive() = TRUE

THEN

IF ExpandCluster(SubImage, Point, ClusterId, Eps, MinPxl)

THEN

ClusterId:=nextId(ClusterId)

**Figure 2:**
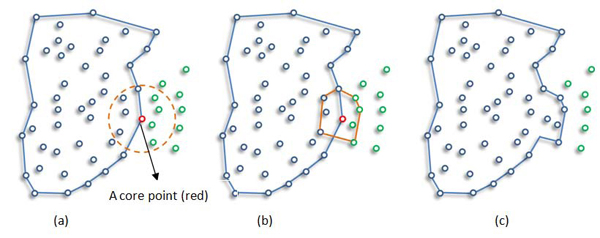
Example Cluster Expanding: New points (green ones in circle) are expanding cluster.

END IF;

END IF;

END FOR;

END FOR;

END DBSCAN;

Algorithm 1 summarizes DBSCAN for image segmentation. Once the two parameters Eps and MinPxl are defined, DBSCAN starts to cluster data points from an arbitrary point q. It begins by finding the neighborhood of point q, i.e., all points that are directly density reachable from point q. This neighborhood search is called region query. For an image, we start with left-top pixel (not necessarily a corner pixel, any arbitrary pixel can be chosen for first iteration) as our first point in the dataset (subimage). We look for first pixel satisfying the core pixel condition as a starting (seed) point. If the neighborhood is sparsely populated, i.e. it has fewer than MinPxl points, then point q is labeled as a noise. Otherwise, a cluster is initiated and all points in neighborhood of point q are marked by new cluster's ID. Next the neighborhoods of all q's neighbors are examined iteratively to check if they can be added into the cluster. If a cluster cannot be expanded any more, DBSCAN chooses another arbitrary unlabeled point and repeats processes to form another cluster. This procedure is iterated until all data points in the dataset have been labeled as noise or with a cluster ID. Figure 2 illustrates example cluster expansion. 

## Fuzzy c-means clustering

Clustering, a major area of study in the scope of unsupervised learning, deals with recognizing meaningful groups of similar items.  Under the influence of fuzzy logic, fuzzy clustering assigns each point with a degree of belonging to clusters, instead of belonging to exactly one cluster. 

In fuzzy event modeling, pixel colors in a dermoscopy image can be viewed as probability space where the pixels with some colors can belong partially to the background class and/or the skin lesion. The main advantage of this method is that, it does not require a priori knowledge about number of objects in the image.

Fuzzy C-Means (FCM) clustering algorithm [34,35] is one of the most popular fuzzy clustering algorithms. FCM is based on minimization of the objective function *F_m_*(*u*, *c*) [35]:

FCM computes the membership *u_ij_* and the cluster centers *c_j_* by:

where *m, the fuzzification factor* which is a weighting exponent on each fuzzy membership, is any real number greater than 1, *u_ij_* is the degree of membership of *x_i_* in the cluster *j*, *x_i_*  is the *i*^th^  of d-dimensional measured data, *c_j_* is the dimension center of the cluster, *d^2^(x_k_,c_i_)* is a distance measure between object x_k_ and cluster center c_i,_ and ||*|| is any norm expressing the similarity between any measured data and the center.  

The FCM algorithm involves the following steps:

1. Set values for c and *m*

2. Initial membership matrix U= [*u_ij_*], which is U^(0)^  (|i| = number of members, |j| = number of clusters)

3. At *k-step:* calculate the centroids for each cluster through equation (2) if k ≠ 0. (If k=0, initial centroids location by random)

4. For each member, calculate membership degree by equation (1) and store the     information in U^(k)^

5. If the difference between U^(k)^ and U^(k+1)^ less than a certain threshold, then STOP;     otherwise, return to step 3.

**Figure 3:**
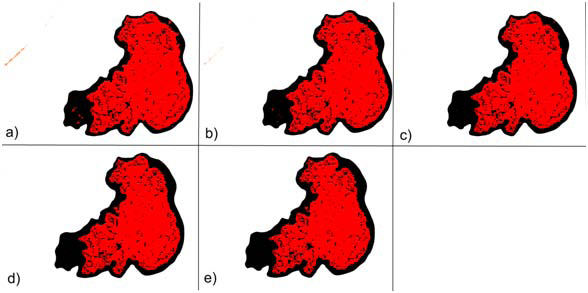
Overlay images of FCM with different threshold values a) 30, b) 40, c) 50, d) 60, e) 70.

In the FCM, the number of classes (*c* in equation 1) is a user input. We tried to find the number of unclassified data points is greater than some threshold (T) values (30, 40, 50, 60, and 70) in our experiments. Since the number of classes is a user input in FCM, there is a risk of over segmentation. For instance when the number of segments in a skin image is 3 and we force the number of clusters to be found by FCM to be 6, the FCM over segments the image. This was one of the principal challenges we encountered with FCM. Thus, we ran FCM for different number of clusters and different threshold values and found that for the value of five initial clusters and threshold value of 30, FCM gave good accuracy in segmentation. Therefore, we used these values in all of our experiments. Moreover, in all of our experiments fuzzification factor *m* is taken as 2. Figure 3 shows how FCM detected area (red region) is changed by the change in threshold.

## Experiments and results

The proposed methods are tested on a set of 100 dermoscopy images obtained from the EDRA Interactive Atlas of Dermoscopy [12]. These are 24-bit RGB color images with dimensions ranging from 577 × 397 pixels to 1921 × 1285 pixels. The benign lesions include nevocellular nevi and dysplastic nevi. The distance function used is Euclidean distance between pixels p and q, and given as  where  p.x and p.y denote position of pixel p at xth column and yth row with respect to top-left corner (0, 0) of  image. We run DBSCAN on each image with the eps of 5 and MinPts of 60. 

**Figure 4:**
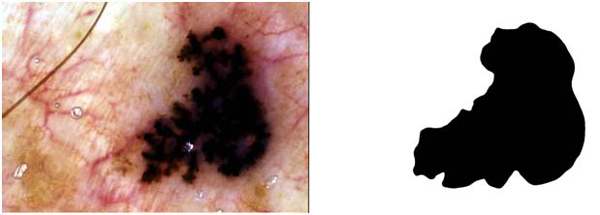
An exemplary dermoscopy image (left) and corresponding dermatologist drawn border (right)

**Figure 5:**
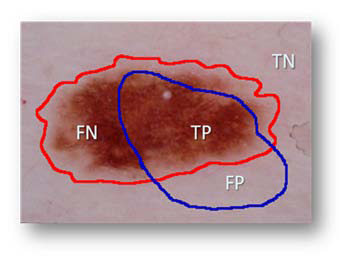
Illustration of components used in accuracy and error quantification.

We evaluated the border detection errors of the DBSCAN and FCM by comparing our results with physician-drawn boundaries as a ground truth. Manual borders were obtained by selecting a number of points on the lesion border, connecting these points by a second-order B-spline and finally filling the resulting closed curve [22]. Using the dermatologist-determined borders, the automatic borders obtained from the DBSCAN and FCM are compared using three quantitative error metrics: border error, precision, and recall. Border error is developed by Hance et al. [18] and currently the most important metric for assessing quality of any automatic border detection algorithm, and given by:

where AutomaticBorder is the binary image obtained from DBSCAN or FCM, ManualBorder is the binary image obtained from a dermatologist (see Figure 4 right side). Exclusive OR operator,⊕, essentially emphasizes disagreement between target (ManualBorder) and predicted (AutomaticBorder) regions. Referring to information retrieval terminology, numerator of the border error means summation of False Positive (FP) and False Negative (FN). The denominator is obtained by adding True Positive (TP) to False Negatives (FN). An illustrative example is given in Figure 5.

Regarding the image in Figure 5, assume that red is drawn by a dermatologist and blue is the automated line, respectively. TP indicates correct lesion region found automatically. Similarly, TN shows healthy region (background) both manual and computer assessment agree on. FN and FP are labels for missed lesion and erroneous positive regions, respectively. Addition to border error, we also reported precision (positive predictive value) and recall (sensitivity) for each experimental image in Table 1 and Table 2 for results generated with DBSCAN and FCM respectively. Precision and recall are defined as  respectively.

**Table 1:** DBSCAN border error, precision, and recall measures for each image in the dataset

Img. ID	Border Error	Precision	Recall		Img. ID	Border Error	Precision	Recall
1	8.2%	0.98	0.79		51	5.1%	1.00	0.81
2	8.0%	0.93	0.86		52	6.9%	1.00	0.80
3	4.9%	0.89	0.85		53	7.4%	1.00	0.78
4	6.2%	1.00	0.82		54	1.5%	1.00	0.95
5	5.4%	1.00	0.88		55	4.2%	1.00	0.88
6	4.6%	1.00	0.83		56	14.9%	1.00	0.60
7	3.9%	0.96	0.91		57	9.4%	1.00	0.77
8	3.2%	1.00	0.87		58	5.9%	1.00	0.82
9	3.4%	1.00	0.82		59	4.6%	1.00	0.75
10	2.2%	1.00	0.91		60	2.9%	1.00	0.81
11	0.9%	1.00	0.91		61	6.5%	0.90	0.74
12	6.5%	1.00	0.61		62	5.8%	1.00	0.74
13	10.0%	1.00	0.70		63	5.6%	1.00	0.77
14	14.8%	1.00	0.70		64	2.9%	1.00	0.82
15	5.9%	1.00	0.67		65	2.2%	0.94	0.84
16	6.8%	1.00	0.76		66	8.3%	0.89	0.79
17	6.0%	1.00	0.67		67	6.3%	0.98	0.83
18	4.0%	1.00	0.86		68	3.2%	1.00	0.79
19	6.4%	1.00	0.71		69	2.4%	1.00	0.79
20	8.0%	1.00	0.80		70	4.6%	1.00	0.74
21	8.8%	1.00	0.78		71	8.8%	1.00	0.71
22	12.6%	1.00	0.73		72	3.5%	0.94	0.84
23	8.6%	1.00	0.76		73	1.8%	0.99	0.86
24	9.0%	1.00	0.72		74	2.9%	1.00	0.90
25	5.7%	1.00	0.79		75	5.9%	1.00	0.71
26	33.9%	1.00	0.51		76	9.2%	1.00	0.74
27	9.0%	1.00	0.74		77	3.3%	1.00	0.72
28	8.0%	1.00	0.65		78	13.6%	1.00	0.61
29	10.6%	1.00	0.75		79	10.4%	1.00	0.71
30	11.3%	1.00	0.74		80	6.7%	1.00	0.65
31	9.7%	1.00	0.72		81	1.8%	1.00	0.65
32	10.8%	1.00	0.77		82	7.5%	1.00	0.82
33	3.3%	1.00	0.86		83	9.9%	1.00	0.54
34	4.2%	1.00	0.88		84	3.1%	1.00	0.74
35	2.7%	1.00	0.88		85	6.4%	1.00	0.79
36	6.0%	1.00	0.79		86	7.5%	0.98	0.79
37	4.0%	1.00	0.85		87	7.2%	1.00	0.73
38	8.0%	1.00	0.71		88	5.1%	1.00	0.59
39	3.4%	1.00	0.76		89	5.5%	0.91	0.82
40	3.6%	1.00	0.82		90	17.0%	1.00	0.56
41	8.0%	1.00	0.73		91	8.1%	1.00	0.61
42	3.2%	1.00	0.85		92	4.3%	1.00	0.89
43	7.3%	1.00	0.74		93	1.7%	1.00	0.93
44	17.7%	1.00	0.70		94	14.6%	1.00	0.66
45	3.6%	1.00	0.84		95	3.0%	1.00	0.68
46	5.2%	1.00	0.88		96	7.8%	1.00	0.75
47	2.5%	1.00	0.91		97	21.8%	1.00	0.66
48	3.0%	1.00	0.87		98	4.0%	1.00	0.85
49	10.9%	1.00	0.68		99	11.5%	1.00	0.65
50	12.0%	1.00	0.68		100	3.1%	1.00	0.66

**Table 2:** FCM border error, precision, and recall measures for each image in the dataset

Img. ID	Border Error	Precision	Recall		Img. ID	Border Error	Precision	Recall
1	99%	1	0.635		51	99.9%	0.99	0.66
2	99.98%	1	0.65		52	132.5%	1	0.5
3	100%	1	0.62		53	69.93%	1	0.45
4	101%	1	0.54		54	100%	1	0.56
5	98%	1	0.66		55	89.47%	1	0.45
6	96%	1	0.55		56	108.13%	1	0.52
7	105%	1	0.645		57	96%	0.99	0.65
8	100%	1	0.66		58	105%	1	0.62
9	89%	1	0.7		59	100%	1	0.56
10	106%	1	0.7		60	78.32%	1	0.51
11	100%	1	0.79		61	96.82%	1	0.53
12	98%	1	0.35		62	106.83%	1	0.34
13	97%	1	0.45		63	100%	1	0.71
14	99%	1	0.76		64	103.33%	0.98	0.56
15	103%	1	0.23		65	101%	1	0.47
16	98%	1	0.63		66	96.86%	0.95	0.52
17	100%	1	0.2		67	100%	1	0.65
18	89%	1	0.54		68	106.83%	1	0.62
19	99%	1	0.33		69	99%	1	0.34
20	99.9%	1	0.67		70	106.67%	1	0.49
21	92.9%	1	0.65		71	102.3%	1	0.65
22	98%	1	0.71		72	99.9%	1	0.71
23	78.3%	1	0.56		73	123%	1	0.48
24	96.8%	1	0.45		74	105.3%	1	0.53
25	106%	1	0.5		75	103.6%	1	0.5
26	123%	1	0.65		76	98%	1	0.58
27	105.4%	1	0.56		77	106.8%	1	0.45
28	104.7%	1	0.59		78	107%	1	0.76
29	98%	0.99	0.501		79	89.3%	1	0.69
30	95%	1	0.63		80	96.8%	1	0.59
31	93.7%	1	0.34		81	100%	1	0.63
32	96.8%	1	0.49		82	102.3%	1	0.34
33	100%	1	0.53		83	103.3%	1	0.56
34	101%	1	0.43		84	100%	1	0.32
35	98%	1	0.39		85	100%	1	0.67
36	103%	1	0.65		86	89%	1	0.65
37	98%	1	0.62		87	106.6%	1	0.71
38	100%	1	0.6		88	99%	1	0.56
39	89%	1	0.46		89	106.8%	0.97	0.5
40	106.6%	1	0.48		90	118.4%	1	0.48
41	93.6%	1	0.54		91	98.3%	1	0.65
42	96.8%	1	0.59		92	99.6%	1	0.62
43	100%	1	0.57		93	122.4%	1	0.45
44	89%	1	0.63		94	100%	1	0.48
45	107%	1	0.76		95	106.6%	1	0.56
46	89.3%	1	0.64		96	93.6%	1	0.43
47	96.8%	1	0.45		97	96.8%	0.99	0.51
48	106.7%	1	0.48		98	100%	1	0.53
49	99%	1	0.56		99	89%	1	0.49
50	99.9%	1	0.59		100	107%	1	0.53

Note that all definitions runs for the number of pixels in the particular region. Analogously, Area() function returns the number of active pixels in a binary image.  Table 1 gives border error, precision and recall rates generated from the DBSCAN for each image whereas Table 2 represents border error, precision and recall rates generated from the FCM. It can be seen that the results vary significantly across the images. 

**Figure 6:**
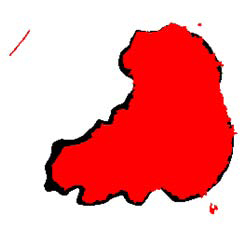
Overlay image of DBSCAN.

In Figure 4, an exemplary dermoscopy image, which is determined as melanoma, and its corresponding dermatologist drawn border are illustrated. Figure 6 illustrates the DBSCAN generated result in red color for the same image. The DBSCAN generated result is overlaid on top of the dermatologist drawn border image in black color. As seen from the figure, hair is detected as false positive. Figure 3 shows results generated from the FCM with different fuzzification factors.

For example for the melanoma image given in Figure 4, FCM’s precision, recall, and border error rates are 99.4%, 75.4%, and 100.4% respectively; however, DBSCAN’s precision, recall, and border error rates for the same image are 94%, 84%, and 2.2% respectively. Following tables show results generated with the DBSCAN and the FCM for 100 image dataset respectively.

**Figure 7:**
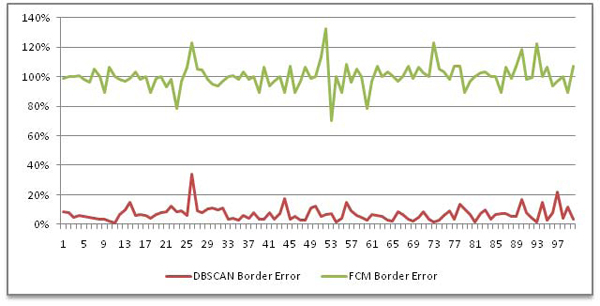
Border errors generated by DBSCAN (red) and FCM (green)

Since the most important metric to evaluate performance of a lesion detection algorithm is border error metric, border errors for DBSCAN and FCM are illustrated in Figure 7. In the figure, X-axis show image IDs in random order. As seen from Figure 7, DBSCAN outperforms FCM for lesion border detection on dermoscopy images: for DBSCAN overall average border error ratio is 6.94% whereas overall average border error ratio for FCM is 100%. As for recall and precision, DBSCAN and FCM averaged out 76.66% and 99.26%; 55% and 100% , respectively. 

**Figure 8:**
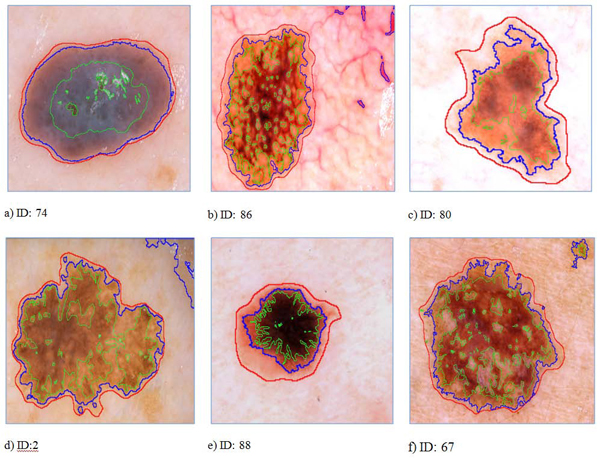
Sample images showing assessments of the dermatologist (red), automated frameworks DBSCAN (blue) and FCM (green) introduced in this study.

Automatically drawn boundaries usually found at more intense regions of a lesion (see Figure 8a, 8b, 8c, 8e, 8f) having promising assessment with DBSCAN. In Figure 8(d) , the DBSCAN marked also outer regions. Obviously, the gradient region between blue and red boundaries seems to be a major problem for the DBSCAN. We believe that even though inter-dermatologist agreement on manual borders is not perfect, most dermatologists will draw borders approximately the red borders as shown in images of Figure 8. This is because the reddish area just outside the obvious tumor border is part of the lesion. 

We also made a rough comparison of the DBSCAN with prior state of the art lesion border detection methods proposed by Celebi et. al 2008 [22] and 2009 [17]. Comparisons showed that the mean error of DBSCAN (6.94%) is obviously less than their results. However, we cannot make image by image comparison since they used a subset of 100 dermoscopy image dataset (90 images). Their image IDs might be different than our image IDs even for the same image. Therefore, for now the mean error rate is only indication we have as a proof that DBSCAN is better than studies given in [17] and [22].

## Conclusion

In this study, we introduced two approaches for automatic detection of skin lesions. First, a fast density based algorithm DBSCAN is introduced for dermoscopy imaging. Second, the FCM is used for lesion border detection. The assessments obtained from both methods are quantitatively analyzed over three accuracy measures: border error, precision, and recall. As well as low border error, high precision and recall, visual outcome showed that the DBSCAN effectively delineated targeted lesion, and has bright future; however, the FCM had poor performance especially in border error metric. The next step, we will focus on at more details on intra-variability and post-assessment during performance analysis of the intelligent systems. Additionally, performance of DBSCAN will be evaluated over different polygon-unioning algorithms. In terms of border errors, we plan to develop model that are more sensitive to melanoma lesion. A thresholding method which is well-integrated with clustering rationale, such as the one described in [36], will be preferred in the future because of unexpected difference between precision and recall rates.

## Competing interests

The authors declare that they have no competing interests in regards to this study.

## Authors' contributions

SK and MM have made equal contributions to this study. Both of the authors participated in the overall design of the study. MM designed the density-based algorithms. SK developed the general comparison testbed, performed data analysis, algorithm testing, statistical measurements, and benchmarking. BB designed and implemented the FCM. KA integrated it to the general framework. SK and MM contributed to the writing of this manuscript. All of the authors read and approved the final manuscript.
